# At the tip: novel regulators of shoot apical meristem development in canola

**DOI:** 10.1093/plphys/kiag126

**Published:** 2026-03-02

**Authors:** Neeta Lohani

**Affiliations:** Assistant Features Editor, Plant Physiology, American Society of Plant Biologists; Department of Biotechnology, Thapar Institute for Engineering and Technology, Patiala, Punjab 147004, India

Meristems produce all plant organs and are key determinants of crop architecture and yield. The shoot apical meristem (SAM), positioned at the growing shoot tip, contains a self-renewing pool of pluripotent stem cells that generate all aerial structures, including leaves, stems, and flowers. Within the SAM, stem cells reside in a stem cell niche organized into functionally distinct zones: the central zone harbors the stem cell reservoir, the organizing center located below the central zone coordinates meristem activity, and the peripheral zone is where new organs initiate. Maintaining appropriate stem cell number requires precise regulatory networks that balance self-renewal with differentiation.

In Arabidopsis, the CLAVATA-WUSCHEL (CLV-WUS) signaling pathway controls the number of stem cells and meristem size. WUS, a homeodomain transcription factor, is transcribed in the organizing center (Mayer et al. 1998). The WUS protein then migrates through plasmodesmata into adjacent central zone cells, where it directly binds the *CLV3* promoter to activate *CLV3* expression (Yadav et al. 2011). This is the key step in the feedback loop: the CLV3 peptide, secreted by stem cells, is perceived by the receptor kinase CLV1 and related receptors, which in turn restrict *WUS* transcription, thereby maintaining stem cell homeostasis (Clark, Williams and Meyerowitz 1997; Fletcher et al. 1999; Schoof et al. 2000). Core elements of the CLV-WUS pathway are conserved across flowering plants, yet identifying the precise pathway components and understanding their functional roles in polyploid crops remains challenging due to extensive gene redundancy. *Brassica napus* (canola/rapeseed), the world's second most important oilseed crop, arose through recent allopolyploidy and carries multiple copies of most genes. The genetic redundancy often masks mutant phenotypes, making it difficult to identify novel meristem regulators or manipulate SAM activity for crop improvement.

In this issue of *Plant Physiology*, Yu et al. (2026) identified novel regulators of SAM development in *B. napus* by integrating transcriptomic profiling with high-throughput CRISPR/Cas9 functional screening. They discovered that SERINE CARBOXYPEPTIDASE-LIKE (SCPL) proteins are upstream regulators of the CLV3-WUS pathway. Plants with mutations in *BnaSCPL29*, *BnaSCPL44*, and *BnaSCPL45* produce multiple main stems associated with disrupted shoot apex organization. Their findings expand our understanding of SAM biology and provide potential targets for engineering crop architecture.

The authors used the existing *Bnaclv1*, *Bnaclv2*, and *Bnaclv3* mutants (Yang et al. 2018), which exhibit multilocular siliques due to enlarged meristems, as an entry point for transcriptomic analysis. By profiling gene expression across 3 developmental stages—inflorescence meristem, flower bud stage 6, and stage 8—they captured the transcriptional changes accompanying SAM phase transitions. Differentially expressed genes among the analyzed stages had minimal overlap, confirming that these represent distinct developmental windows with unique transcriptional programs.

Weighted gene coexpression network analysis was used to prioritize candidate genes for SAM regulation. Their analysis identified a module containing 237 genes that showed a strong correlation with the multilocular trait, which is the characteristic phenotype of *clv* mutants (*r* = 0.8). From these, 45 genes in *B. napus*, corresponding to 44 *Arabidopsis thaliana* orthologs, exhibited consistent differential expression across multiple comparisons and clustered around *CLV3* as a central hub in the coexpression network. Excluding *CLV3* and *STM*, whose SAM roles were previously established, the analysis yielded 42 candidates for further functional analysis. Notably, several candidates, including *SCPL29*, *SCPL44*, *SCPL45*, and *OTU9*, had no previously documented connection to SAM function in either *B. napus* or *A. thaliana*.

Using a high-throughput multiplex CRISPR/Cas9 pipeline (He et al. 2023), Yu et al. simultaneously targeted the 42 candidate genes across their homeologous copies in *B. napus*, successfully generating mutants for 25 genes, including homozygous knockouts for 9 of them. This is a significant achievement given the genomic complexity of this polyploid crop.

The phenotypic consequences of *BnaSCPL* mutations were striking (Fig. 1a). Homozygous *Bnascpl29*, *Bnascpl44*, and *Bnascpl45* mutants all developed multiple main stems of comparable thickness and height, contrasting with the single-stemmed wild-type architecture. *Bnascpl* mutants developed multiple meristematic structures at the shoot apex by day 7 after germination, and the phenotypes became more pronounced at day 14 (Fig. 1a). Wild-type plants possessed a single SAM positioned between the leaf stalks, whereas SCPL mutants displayed multiple meristematic structures. The *Bnascpl29* mutant exhibited well-separated, independent meristems, whereas *Bnascpl45* showed closely adjacent structures. The *Bnascpl44 Bnascpl45* double mutant presented the most severe phenotype, with irregular lobules and protruding tissue at the shoot apex. These distinct phenotypes suggest functional specialization within the Class II SCPL family despite their phylogenetic proximity. Importantly, the authors note that whether the multiple main stems originate from SAM splitting or from enhanced axillary meristem activity remains to be determined.

**Figure 1:**
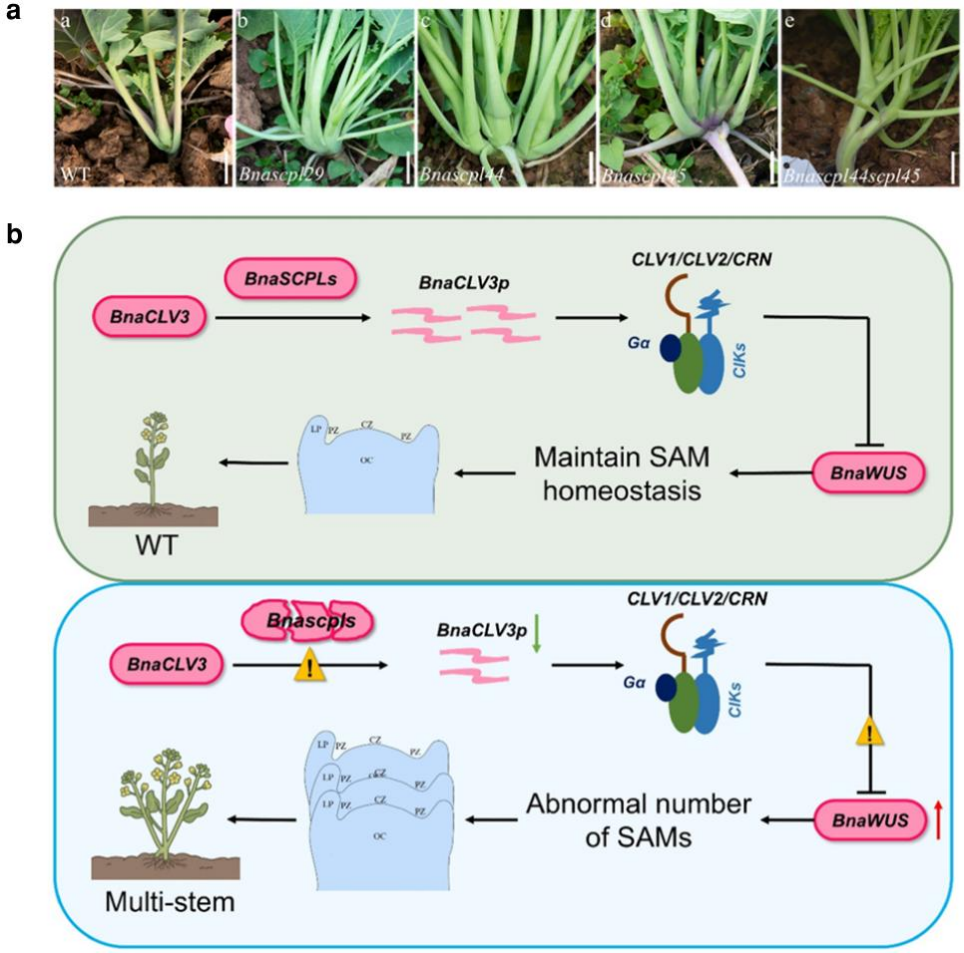
BnaSCPL proteins regulate SAM organization and plant architecture in *Brassica napus*. **a)** Mutations in *BnaSCPL* lead to disruption of SAM organization and plant architecture in *Brassica napus*. a) Field-grown phenotypes of wild-type (WT) and (b, c, d, e) *Bnascpl* mutants. WT displays a single main stem, while *Bnascpl29*, *Bnascpl44*, *Bnascpl45*, and *Bnascpl44Bnascpl45* mutants develop multiple main stems of comparable height. **b)** A proposed model of BnaSCPL-mediated regulation of meristem development. In WT plants, BnaSCPLs process the CLV3 precursor into mature peptide, which signals through CLV1/CLV2/CRN receptors to repress *WUS*, maintaining SAM homeostasis. In *scpl* mutants, impaired processing reduces the concentration of mature CLV3 peptide, weakening repression of *WUS* gene expression and leading to abnormal SAM numbers and a multi-stem phenotype. Adapted from Yu et al. (2026).

Molecular characterization revealed that *BnaSCPL* mutations disrupt the CLV3-WUS regulatory circuit. In *Bnascpl44* and *Bnascpl45* single mutants, both *BnaCLV3* and *BnaWUS* expression was significantly elevated, accompanied by upregulation of receptor complex components, including *BnaCLV1*, *BnaCLV2*, and *BnaCRN*. The authors propose that BnaSCPLs process the CLV3 precursor protein into its bioactive peptide form, a critical step in CLE peptide maturation (Ni and Clark 2006). In mutants lacking functional SCPLs, reduced mature CLV3 peptide weakens receptor-mediated suppression of *WUS*, leading to expansion of the *WUS* expression domain. The elevated *BnaCLV3* transcription likely represents a compensatory feedback response, while receptor upregulation may enhance sensitivity to the diminished peptide signal.

An interesting finding emerged from the *Bnascpl44 Bnascpl45* double-mutant analysis. Despite exhibiting the most severe meristematic phenotype, the double mutants showed no significant upregulation of *BnaCLV2*, *BnaCLV3*, or *BnaCIK1* relative to wild type, even though the *BnaWUS* expression domain remained expanded. This phenotypic reversion in receptor expression suggests that BnaSCPL44 and BnaSCPL45 have distinct yet interdependent roles. The authors hypothesize that BnaSCPL44 may provide partial functional compensation when BnaSCPL45 alone is lost, and simultaneous deletion of both genes disrupts this compensatory network, uncoupling receptor expression from WUS regulation.

The precise mechanism by which BnaSCPLs influence CLV3 signaling remains to be fully determined. Yeast 2-hybrid assays detected no direct interaction between BnaSCPLs and the full-length CLV3 precursor protein, consistent with the hypothesis that SCPLs may act on intermediate processing forms rather than the initial holoprotein. However, the authors acknowledge alternative possibilities. Similar to GS5, a rice SCPL protein that modulates grain size by binding the extracellular leucine-rich repeat domain of OsBAK1-7 (Xu et al. 2015), BnaSCPLs might interact directly with CLV pathway receptors to modulate signaling strength. Distinguishing between these mechanisms will require in vitro cleavage assays with synthetic CLV3 peptides at various processing stages, alongside receptor interaction studies.

Beyond *SCPL* genes, this study also provides insights into LEAFY (*LFY*) function in *B. napus*. LFY is a key transcription factor controlling floral meristem identity. Triple homozygous *Bnalfy* mutants exhibited a complete failure to initiate flowers, entering a permanent vegetative state that continuously produced leaf buds. Functional divergence was evident among homeologs: loss of *BnaCnn-2.LFY* alone caused abnormal flowers with sepal deletion and reduced pollen, while single mutations in *BnaA06.LFY* or *BnaCnn-1.LFY* produced no phenotypic differences from wild type. Notably, *BnaCnn-2.LFY* has the lowest expression among the three homeologs, yet is functionally indispensable, demonstrating that expression abundance does not necessarily predict functional importance in polyploids.

This study establishes BnaSCPLs as novel upstream regulators of the CLV3-WUS pathway affecting plant architecture (Fig. 1b). While SCPLs have been implicated in reproductive development—GS5 and its homologs modulate grain size in cereals (Xu et al. 2015), and AtSCPL22 overexpression increases carpel number in Arabidopsis (Wen, Li and Walker 2012)—the finding that specific SCPL family members control branching architecture represents a significant expansion of our understanding of the roles of this protein family in development. From an applied perspective, the multi-stem phenotype of *scpl* mutants offers possibilities for optimizing canopy architecture, light interception, and resource allocation in crop production systems. The high-throughput CRISPR pipeline developed here provides a template for functional genomics in polyploid species where gene redundancy typically masks phenotypic effects. As climate change demands increasingly sophisticated approaches to crop improvement, engineering SAM biology through newly identified regulators like SCPLs offers promising avenues for architectural optimization.

## Recent research articles in *Plant Physiology*:

- Gregory et al. (2024) reported that REL2-RELK co-repressors fine-tune hormone and redox cues to prevent expanded expression of *ZmWUSCHEL1* in maize inflorescence meristems and can potentially be harnessed for increasing seed yield in hybrids (https://doi.org/10.1093/plphys/kiae476)
- Wang et al. (2024) provided evidence that the heterozygous mutations in the CLV-WUS pathway genes *ZmCRN* and *ZmCLE7* subtly enlarge the maize inflorescence meristem, increasing kernel row number and yield in hybrid lines without detrimental pleiotropic effects (https://doi.org/10.1093/plphys/kiae472)

## Data Availability

No new data were generated or analyzed in support of this article.
